# Evaluation of the effect of dose-dependent platelet-rich fibrin membrane on treatment of gingival recession: a randomized, controlled clinical trial

**DOI:** 10.1590/1678-7757-2017-0278

**Published:** 2018-05-03

**Authors:** Rana Culhaoglu, Levent Taner, Berceste Guler

**Affiliations:** 1 Oral and Dental Health Center, Kırıkkale, Turkey; 2 Gazi University, Faculty of Dentistry, Department of Periodontology, Ankara, Turkey; 3 Dumlupınar University, Faculty of Dentistry, Department of Periodontology, Kütahya, Turkey

**Keywords:** Gingival recession, Periodontics, Regeneration

## Abstract

Miller's class I gingival recessions (GR) have been treated using coronally advanced flap (CAF) with platelet-rich fibrin membrane (PRF membrane) or connective tissue graft (CTG).

**Objective:**

The aim of this study was to evaluate the effect of different multiple layers of PRF membranes for the treatment of GR compared with the CTG procedure.

**Material and Methods:**

Sixty-three Miller class I GR were treated in this study. Twenty-one GR selected randomly were treated with two layers of PRF membranes+CAF in 2PRF+CAF (test group-1), four layers of PRF membranes+CAF in 4PRF+CAF (test group-2), and CTG+CAF in the control group. The plaque index (PI), gingival index (GI), probing depth (PD), keratinized tissue thickness (KTT), clinical attachment level (CAL), recession depth (RD), recession width (RW), and keratinized tissue height (KTH) measurements were performed at baseline and 1, 3, and 6 months after surgery. The post-operative discomfort of patients, assessed with the visual analog scale (VAS) and healing index (HI), was recorded after surgery.

**Results:**

PI, GI, and PD scores were similar for all patients at all times. RD and RW scores were similar for each patient at 1 month, but these values were significantly increased in the subsequent periods in test group-1. The increase in KTT was significantly higher in the control group compared with the test groups. Similar root coverage scores were obtained in the test group-2 and control groups, and these scores were significantly higher compared with test group-1.

**Conclusions:**

The PRF membrane+CAF technique may be an alternative to the CTG+CAF technique for postoperative patient comfort. However, PRF membranes should use as many layers as possible.

## Introduction

Gingival recession (GR) is the apical migration of the marginal tissue beyond the cementoenamel junction (CEJ)^29^. GR occurs because of traumatic tooth brushing, excessive frenulum attachments, and malposition of teeth. In addition, it may result in dentinal hypersensitivity, root caries, and unaesthetic gingival appearance^25^ ^–^ ^29^. There are various techniques that provide coverage of the exposed roots^3^. The primary purposes of mucogingival surgeries are complete root coverage (CRC), pleasant aesthetic outcomes, and minimal probing depth after surgery^25^ ^–^ ^29^.

Multiple GR were successfully covered using the envelope type of coronally advanced flap (CAF) technique^25^ ^,^ ^28^. This procedure encompasses sliding the soft tissues onto a recession defect when there is existing adequate keratinized tissue^25^ ^,^ ^28^. The CAF technique is used to treat both single^21^ ^,^ ^28^ and multiple recessions^3^ ^,^ ^21^. Moreover, CAF is effective in the treatment of multiple recessions affecting adjacent teeth, with clear advantages for the patient^21^ ^,^ ^25^.

A coronally advanced flap has been used in combination with various techniques to increase coverage and obtain CRC^5^ ^,^ ^20^. CAF+connective tissue grafts and CAF+platelet concentrated grafts are some of these procedures^5^.

A connective tissue graft (CTG) combined with CAF (bilaminar technique) is considered the gold standard for Miller Class I and II recession defects^13^ ^,^ ^25^ and recent studies have reported that the percent root coverage (RC) ranges from 79.9% to 89.6%^4^ ^,^ ^9^ ^,^ ^13^. The advantage of this technique is the enhancement of keratinized tissue width, which can be explained by the determination of CTG surface epithelium characteristics^13^. Moreover, there are many disadvantages, such as postoperative pain or bleeding and a second surgical site after CTG operations^28^.

Platelet-rich fibrin (PRF) is the second generation of platelet concentrates^7^. A PRF membrane contains many growth factors, such as platelet-derived growth factor (PDGF), which is effective in the soft tissue wound healing process because it stimulates angiogenesis, granulation tissue formation, and epithelial migration. These substances have been used together with GR treatment techniques, but there are limited data about factors affecting root coverage in human studies^9^ ^,^ ^24^.

Therefore, the aim of this study was to evaluate the different amounts of PRF membranes for the treatment of Miller Class I gingival recessions compared with the CTG procedure, considered the gold standard.

## Material and methods

Twenty-two subjects, 10 males and 12 females, aged from 21 to 52 years with gingival recession complaints were included the study. Patients from the Department of Periodontology at the Gazi University, Ankara, were enrolled from April 2014 to February 2015. Ethical permission was obtained from the Ethical Committee of Ankara University Faculty of Dentistry, Ankara (November 12, 2013, protocol no: 10/3). All patients were instructed about benefits and risks of the study and each participant signed an informed consent form. The trial is registered at ClinicalTrials.gov, number NCT02882464.

### Study population

The inclusion criteria were: 1) multiple Miller Class I gingival recession defects on the lower and/or upper incisors, canines, or premolars; 2) similar tooth numbering of teeth with multiple GR defects, ranging from two to three defects, 3) presence of ≥1 mm keratinized tissue apical to the recession; 4) presence of an identifiable cementoenamel junction (CEJ); 5) ≤2 mm probing depth (PD); and 6) no previous periodontal surgical procedures in the recession area. Exclusion criteria were: 1) smoking; 2) pregnancy; 3) caries, deep abrasion, restoration or pulpal pathology on the teeth involved; and 4) use of steroid, bisphosphonates, or irradiation therapy.

### Study design

This study was a randomized, controlled clinical trial that compared different amounts of PRF membrane layers and CTG for the treatment of Miller Class I gingival recessions. In the 2PRF+CAF, 21 teeth with GR defects were treated with two layers of PRF membranes+CAF and the group was defined as 2PRF+CAF; in the 4PRF+CAF, 21 teeth with GR defects were treated with four layers of PRF membranes+CAF and the group was defined as 4PRF+CAF; and in the control group, 21 teeth with GR defects were treated with CTG+CAF.

### Randomization

Subjects were assigned to one of the three groups using a computer-generated randomization scheme. Allocation concealment was obtained using numberlabeled opaque envelopes that were opened just before the surgery.

### Sample size

A software program (G*Power v. 3.0.10, Los Angeles, CA, USA) was used to calculate the sample size. The power was calculated to be 85% when there were 20 teeth with GR in each group, using an α value of 0.05.

### Clinical measurements

All clinical measurements were performed by one of the examiners. Individual acrylic stents were prepared for all patients to take measurements of the constant points. The following clinical measurements were taken 1 week before surgery (baseline) and at the 1-, 3- and 6-month follow-up visits: 1) recession depth (RD); the distance from the CEJ to the most apical point of the free gingival margin; 2) recession width (RW), which is the horizontal distance of the recession in the mesiodistal direction at CEJ; and 3) keratinized tissue height (KTH), which is the distance from the mucogingival junction to free gingival margin. The following clinical measurements were taken 1 week before surgery and at the 3- and 6-months follow-up visits: 1) plaque index (PI); 2) gingival index (GI); 3) probing depth (PD); 4) keratinized tissue thickness (KTT), which was measured under local anesthesia at the mid-point location between the gingival margin and mucogingival junction using a spreader, and its stopper silicone disc and penetration depth was measured using a standardized caliper^9^; 5) clinical attachment level (CAL); and 6) root coverage (RC) was calculated in millimeters and percentages according to the following formula: [(pre-operative RD − post operative RD)/pre-operative RD] × 100. Standardized photographs were taken at baseline and at 1, 3, and 6 months after surgery. Post-operative discomfort and bleeding were recorded using a visual analogue scale (VAS). The patients recorded their pain level for the first 7 days after surgery on a horizontal scale, where the left endpoint meant no pain (0), the middle point meant pain (50), and the right endpoint meant severe pain (100). The amount of analgesic taken by the patients after surgery was recorded.

### Initial therapy

Patients received oral hygiene instructions and initial periodontal therapy, including scaling and professional tooth cleaning using a low abrasive polishing paste. They were instructed to use the “Roll” brushing technique and a soft toothbrush to protect the soft tissue from trauma.

### Preparation of the PRF membrane

Before the surgery, intravenous blood samples (10 ml) were collected from patients in the test groups. In test group-1, 2 tubes of 10 ml venous blood were collected and in the test group-2, 4 tubes of 10 ml venous blood were collected from each patient. Samples were centrifuged at 2700 rpm for 12 minutes (PC-02 machine, Process Ltd., France) and PRF clots were obtained according to the procedure described by Choukroun^7^ ^–^ ^9^. After coagulation, each PRF clot was prepared in fragment or membrane form using a “PRF Box”^7^ ^–^ ^13^.

### Surgical procedure

All surgical procedures were performed by the same examiner. Patients received local anesthesia with 80 mg/2 ml of articaine hydrochloride and 0.02 mg/2 ml of adrenalin (Vem, Turkey) at recession sites in test groups −1 and −2, and at both recession and donor sites in the control group. Chlorhexidine mouthwash (chlorhexidine gluconate, Drogsan, Ankara, Turkey) was used for intraoral disinfection, and antiseptic solution (polyvinylpyrrolidone iodine 10%, Adeka, Turkey) for extraoral disinfection. In all study groups, each tooth had its mucoperiosteal flap incisions extended to the distal and mesial directions from the recession defects, according to the CAF procedure.

**Figure 1:**
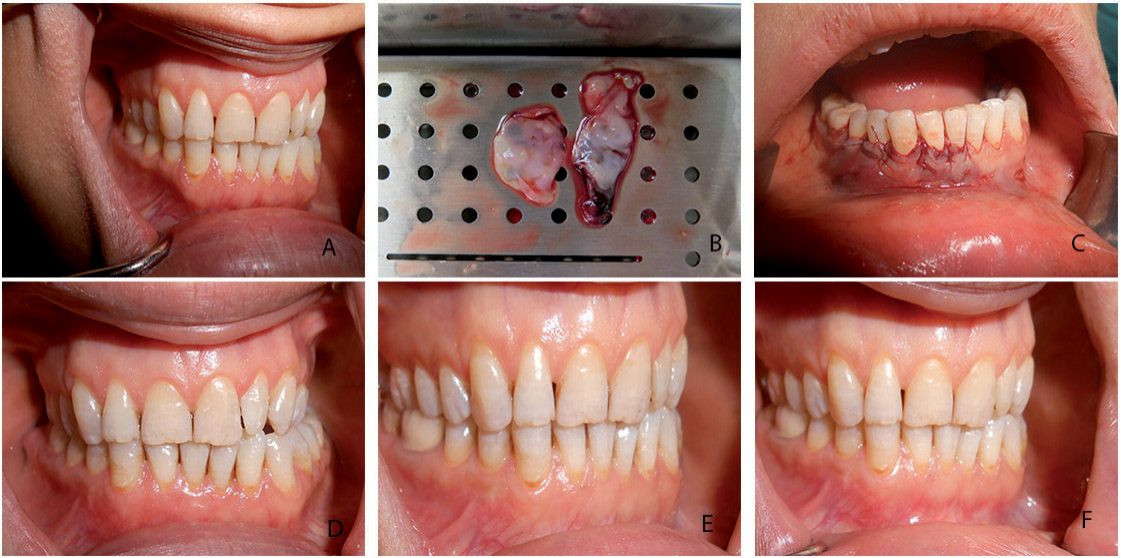
Test group-1 (2 PRF+CAF). a) baseline gingival recession; b) PRF membranes, c) PRF membranes and CAF sutured over the recession; d) 1-month postoperative view; e) 3-month postoperative view, f) 6-month postoperative view

**Figure 2:**
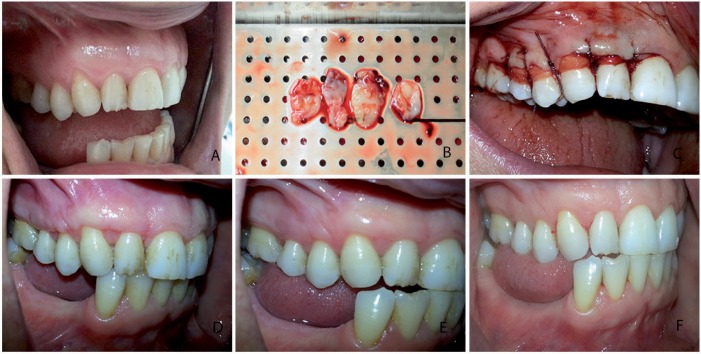
Test group-2 (4 PRF+CAF). a) baseline gingival recession; b) PRF membranes, c) PRF membranes and CAF sutured over the recession; d) 1-month postoperative view; e) 3-month postoperative view; f) 6-month postoperative view

After local anesthesia, in test groups, a horizontal sulcular incision was designed at the buccal side of the recession area at the level of the CEJ, and the incision was extended into the interdental area to be connected with the CEJ. A split thickness flap was raised without vertical incision^2^, the papillae were de-epithelialized, and the root was planned by removing hard accumulations, but no chemical root treatment was performed. In 2PRF+CAF, two layers of PRF membranes were positioned over the recession area at the level of the CEJ (Figure 1). In 4PRF+CAF, four layers of stacked PRF membranes were positioned over the recession area at the level of CEJ (Figure 2). Membranes were sutured to the recipient area using a 6.0 resorbable suture (polyglycolic acid, Doğsan, Turkey) at the level of the CEJ. A split thickness flap was coronally advanced and sutured with a 5.0 resorbable suture (polyglycolic acid, Doğsan, Turkey).

**Figure 3:**
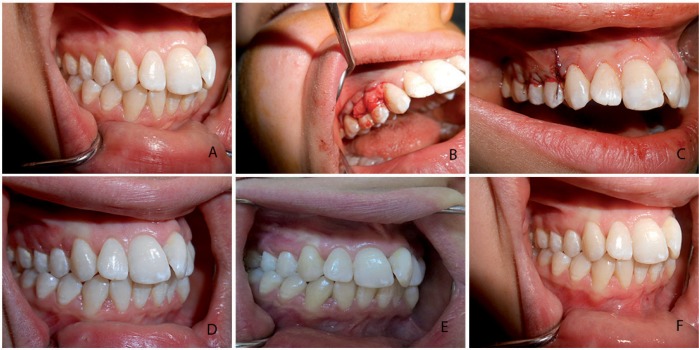
Control group (CTG+CAF). a) baseline gingival recession; b) CTG positioned at the CEJ; c) CTG+CAF sutured over the recession; d) 1-month postoperative view; e) 3-month postoperative view; f) 6-month postoperative view

The surgical technique in the CTG+CAF group was the “envelope technique”, as described by Raetzke^18^ (1985). A horizontal incision at the level of the CEJ was designed and a split thickness flap was raised without any vertical incision. The papillae were de-epithelialized. The root was planned, and hard accumulations were removed with no chemical root treatment performed. The connective tissue graft was harvested from the palate using the “trap-door technique”, described by Edel^10^ (1974). The epithelial layer was elevated with one horizontal and two vertical incisions. The connective tissue graft was harvested at 1 mm using a standard caliper, and the epithelial layer was then sutured using a resorbable suture. The connective tissue graft was sutured to the recipient area using a resorbable suture at the level of the CEJ. A split thickness flap was coronally advanced and sutured using a resorbable suture (Figure 3). Finally, a periodontal dressing was fixed on the recipient's surgical area.

### Postoperative care

Patients were instructed to rinse their mouth twice every day with chlorhexidine solution (chlorhexidine gluconate, Drogsan, Ankara, Turkey) for 1 minute and not to brush their teeth in the surgical area for 2 weeks. Analgesics (flurbiprofen 100 mg, Sanovel, Istanbul, Turkey) were prescribed to be taken only if necessary to control the post-operative pain, and the amount taken was recorded for 1 week. The periodontal dressing was removed 10 days after surgery. Surgical wounds were carefully cleansed with chlorhexidine gluconate, and patients were advised to use soft toothbrushes.

### Statistical analyses

A statistical software program (SPSS version 20.0, Armonk, NY, USA) was used for data analysis. A value of p<0.05 was considered statistically significant. The Friedman's one-way analysis of variance (ANOVA) and the Wilcoxon Sign Test were used to evaluate the intra-group differences and the Kruskal-Wallis H test was used to evaluate the inter-group differences in case the data was not normally distributed. If there was a significant difference in the Kruskal-Wallis H test, a *post-hoc* multiple comparison test was used to determine differences between the groups. Spearman's correlation coefficient was used to evaluate the relationship between data without normal distribution, and Pearson's correlation coefficient was used to evaluate the relationship between data with a normal distribution.

## Results

### Demographic data

There were 63 Miller class I gingival recessions in 22 patients in this study. There were more male patients (76.2%) than females, and the mean age was 37.7±4.1 years in 2PRF+CAF. The number of male and female patients was similar, and the mean age was 45.4±5.0 in 4PRF+CAF. There were more female patients (90.5%) than male patients and the mean age was 31.6±7.2 in the CTG+CAF group. The GR on the maxilla were the most treated in all groups.

### Intergroup comparisons

Clinical evaluation of recession-defect characteristics from baseline and 1, 3, and 6 months after the root coverage surgery are shown in Tables 1 and 2. PI, GI, and PD were similar for all groups and the difference was not statistically significant at baseline, 3, and 6 months after surgery (p>0.05). The reduction in RD was significantly higher in 4PRF+CAF and CTG+CAF groups compared with the 2PRF+CAF group at 3 and 6 months after surgery (p<0.05). Compared with baseline, RD values were reduced in all patients after recession treatments (p>0.05).

**Table 1:** Clinical evaluation of PI, GI, PD, RD, RW

Variable	Test group-1 (n=21)		Test group-2 (n=21)		Control group (n=21)		
	Mean±SD	Median	Mean±SD	Median	Mean±SD	Median	p
PI							
Baseline	0.13±0.13	0.25	0.13±0.13	0.25	0.14±0.13	0.25	0.939
3 months	0.18±0.16	0.25	0.17±0.16	0.25	0.17±0.18	0.25	0.935
6 months	0.31±0.21	0.25	0.30±0.17	0.25	0.35±0.19	0.25	0.725
GI							
Baseline	0.05±0.10	0	0.04±0.09	0	0.05±0.10	0	0.897
3 months	0.12±0.17	0	0.13±0.20	0	0.11±0.15	0	0.994
6 months	0.04±0.09	0	0.02±0.08	0	0.06±0.11	0	0.441
PD							
Baseline	1.45±0.25	1.5	1.37±0.30	1.5	1.31±0.28	1.25	0.218
3 months	1.25±0.24	1.25	1.38±0.25	1.25	1.27±0.25	1.25	0.201
6 months	1.25±0.18	1.25	1.21±0.24	1.25	1.17±0.20	1	0.309
RD							
Baseline	2.71±0.70	3	2.48±0.60	2	2.64±0.57	2.5	0.443
1 month	0.95±0.50	1	0.93±0.48	1	0.90±0.62	1	0.951
3 months	1.24±0.56	1.5	0.74±0.54	0.5	0.74±0.54	0.5	0.004*
6 months	1.67±0.64	1.5	0.81±0.56	0.5	0.52±0.51	0.5	0.0001*
RW							
Baseline	3.62±0.61	4	3.14±0.85	3	3.12±0.57	3	0.031*
1 month	2.86±0.65	3	1.40±0.49	1.5	1.38±0.97	1.5	0.0001*
3 months	2.60±0.68	2.5	1.29±0.41	1.5	1.19±0.68	1.5	0.0001*
6 months	2.64±0.59	3	1.24±0.58	1	1.02±0.81	1	0.0001*

**Table 2:** Clinical evaluation of KTT, KTH, CAL, RC

Variable	Test group-1 (n=21)		Test group-2 (n=21)		Control group (n=21)		
	Mean±SD	Median	Mean±SD	Median	Mean±SD	Median	p
KTT							
Baseline	1.75±0.35	1.73	1.60±0.34	1.52	1.61±0.49	1.62	0.305
3 months	1.98±0.45	1.87	1.81±0.41	1.73	2.39±0.42	2.43	0.0001*
6 months	1.86±0.49	1.75	1.78±0.42	1.62	2.35±1.02	2.47	0.0001*
KTH							
Baseline	4.43±1.21	4	3.95±1.02	4	3.05±0.86	3	0.001*
1 month	4.43±1.08	4	4.00±1.14	4	4.19±0.93	4	0.342
3 months	4.62±0.92	4	3.90±1.09	4	5.05±1.02	5	0.005*
6 months	4.86±0.96	5	4.14±1.24	4	5.29±1.01	5	0.006*
CAL							
Baseline	4.10±1.03	4	3.86±0.69	4	3.88±0.80	4	0.807
3 months	2.52±0.66	2.5	2.21±0.68	2.5	2.02±0.84	1.5	0.081
6 months	2.86±0.74	3	1.93±0.69	2	1.57±0.71	1.5	0.0001*
RC							
1 month	65.57±11.94	66.6	63.07±13.12	60	68.02±20.39	66.6	0.786
3 months	55.35±14.60	50	72.03±15.05	75	72.58±17.11	75	0.001*
6 months	56.34±14.51	50	69.65±15.28	75	80.13±18.93	83.3	0.0001*
RW							
Baseline	3.62±0.61	4	3.14±0.85	3	3.12±0.57	3	0.031*
1 month	2.86±0.65	3	1.40±0.49	1.5	1.38±0.97	1.5	0.0001*
3 months	2.60±0.68	2.5	1.29±0.41	1.5	1.19±0.68	1.5	0.0001*
6 months	2.64±0.59	3	1.24±0.58	1	1.02±0.81	1	0.0001*

RW values were significantly higher in 2PRF+CAF compared with 4PRF+CAF and CTG+CAF groups at all times. The increase in KTT was significantly higher in the CTG+CAF group than in the test groups at 3 and 6 months after surgery (p<0.05).

At 3 months after surgery, KTH values were significantly higher in the CTG+CAF group (5.05±1.02 mm) compared with test groups (4.62±0.92 mm and 3.90±1.09 mm for 2PRF+CAF and 4PRF+CAF, respectively). At 6 months after surgery, KTH values were higher in the 2PRF+CAF (4.86±0.96 mm) and CTG+CAF (5.29±1.01 mm) groups than in the 4PRF+CAF (4.14±1.24 mm) group. However, KTH was significantly higher in 2PRF+CAF than in the other groups at the baseline.

Despite the similarity in the initial CAL value in all groups, the clinical attachment gain was higher in the 4PRF+CAF and CTG+CAF groups than in the 2PRF+CAF group. RC was higher in the 4PRF+CAF and CTG+CAF groups than in the 2PRF+CAF group at 3 and 6 months after surgery. RC was 56.34%, 69.65%, and 80.13% at 6 months for the 2PRF+CAF, 4PRF+CAF and CTG+CAF groups, respectively. Post-operative pain and the amount of analgesic used after surgery were significantly higher in the CTG+CAF group than in the test groups (Table 3).

**Table 3:** Evaluation of patient's post-operative discomfort

Variable	Test group-1 (n=21)		Test group-2 (n=21)		Control group (n=21)		
	Mean±SD	Median	Mean±SD	Median	Mean±SD	Median	p
VAS-1	23.33±15.28	20	19.05±15.78	10	66.19±18.84	60	0.0001*
VAS-2	20.95±11.36	20	18.10±11.67	20	37.14±21.19	40	0.001*
VAS-3	10.00±9.49	10	7.62±11.79	0	25.71±16.30	30	0.0001*
VAS-4	1.90±6.02	0	2.86±9.02	0	35.71±17.48	40	0.0001*
VAS-5	1.43±3.59	0	2.38±4.36	0	25.71±24.76	20	0.0001*
VAS-6	0.00±0.00	0	0.95±3.01	0	11.43±15.82	0	0.0001*
VAS-7	0.00±0.00	0	0.00±0.00	0	6.19±12.03	0	0.005*

### Intragroup comparisons

#### 2PRF+CAF group

PI at 6 months values were significantly higher than at baseline and 3 months after surgery (p<0.05). Significant decreases in RD values were observed at 1, 3, and 6 months after surgery compared to baseline (p<0.05).

RW values were significantly higher at 1, 3, and 6 months after surgery compared with baseline, and at 1 month after surgery compared with 3 months after surgery (p<0.05). At 3 and 6 months after surgery, KTT values were significantly higher than at baseline (p<0.05). However, KTT at 3 months was higher than at 6 months. Additionally, KTH at 6 months after surgery was higher than at baseline and 1 month after surgery.

Clinical attachment gain during the 6-month observation was statistically significant. Compared with the 3 and 6 months subsequent to the RC procedure, the calculated RC values were higher at 1 month after surgery. Each day postoperative pain was significantly reduced compared to the previous day.

#### 4PRF+CAF group

RD values decreased significantly from baseline to 6 months after surgery (p<0.05). Additionally, RD and RW values at 1 month after surgery were significantly higher than RD values at 3 months, but there was no statistically significant difference between the 3- and 6-month periods after surgery. Clinical attachment gain was statistically significant during the 6-month observation (p<0.05). However, CAL at 3 months was higher than at 6 months after surgery. The calculated RC rate was at a similar level for the entire postoperative surgery observation time. Postoperative pain significantly decreased each day compared to the previous day.

#### CTG+CAF group

PI and PD values were similar to the test groups at baseline and postoperative 3^rd^ and 6^th^ months (p<0.05). RD and RW were significantly higher than at 6 months and there was no statistical difference between 3 and 6 months after surgery (p>0.05). Significant increases were observed in the CTG+CAF group compared to the test groups for KTT and KTH. KTT and KTH values from baseline to 6 months increased in the CTG+CAF group and there was no statistical difference between 3 and 6 months after surgery for KTT (p>0.05).

Postoperative pain reduced daily in the first week after the surgery. However, the mean pain level increased on the fourth day after surgery, but the difference between the pain level at 4 days and at 3 days was not statistically significant.

## Discussion

The coronally advanced flap procedure is the most common root coverage surgical technique^3^. Platelet-rich membranes with CAF were used for the treatment of GR in many studies^3^ ^,^ ^13^ ^,^ ^28^. No study has compared the results when different amounts of PRF membranes were used for GR, or no study has clearly indicated the amount of PRF membranes were used.

Clinical measurements were performed in other studies at baseline and 10 days, 1, 3, and 6 months after surgery as in other mucogingival studies^2^ ^,^ ^20^. Histological evaluation of the graft demonstrates that healing and revascularization of the subepithelial connective tissue graft continues for 28–60 days after surgery^12^. Therefore, PI, GI, PD, CAL, and KTT measurements were performed at baseline and 3, 6 months after surgery in our study.

GR are usually seen in people with good oral hygiene. Brushing time, frequency, and the techniques, applied force, bristle hardness, and brush change frequency are factors involved in GR^19^. In this study, patients were instructed to perform a non-traumatic brushing technique (Roll technique) and to use a soft toothbrush^1^.

Initial tissue thickness with >1.2 mm at the attached mucosa and >0.8 mm at the alveolar mucosa was related to the 100% root coverage^27^. In this study, initial mean tissue thickness of the attached mucosa at the level of the gingival sulcus was 1.75 mm for 2PRF+CAF, 1.60 mm for 4PRF+CAF, and 1.61 mm for the control group.

During GR treatment, different flap designs were used. Vertical incisions were performed to enhance flap mobilization in the coronal direction, and more recently, the CAF procedure has been used^17^ ^,^ ^22^. However, vertical incisions reduce the vascularization of the flap from the surrounding soft tissue, and enhance the traumatic surgical area and healing period^3^. In recent studies, the flap was elevated across the adjacent tooth in the apical direction and no vertical incision was performed^2^ ^,^ ^26^. “Langer & Langer” is the most commonly used connective tissue graft technique because it provides more flap repositioning in the coronal direction and covers the graft completely^11^ ^,^ ^27^. In this study, the “envelope” CTG technique was used to standardize the flap design in the test and control groups. Additionally, this procedure provided earlier healing and better aesthetic results on the recipient side.

In a recent study by Öncü^14^ (2017), a similar technique was used to treat multiple recessions with PRF+CAF or CTG+CAF. The more successful RC values were obtained in our study compared to results obtained by Öncü (77.12% and 84% for the PRF+CAF and CTG+CAF groups, respectively). In the outcomes reported by Öncü, KTT values increased significantly in both groups after 6 months. However, KTT at 3 months was higher than at 6 months in all patients in our study. There was no evaluation of measurements on the follow-up held 3 months after surgery in that recent study. The increase in KTT values in CTG+CAF groups were significantly higher than in PRF+CAF groups in our study and these results were similar to those found by Öncü. The author stated that PRF technique has the advantages for patients during surgery and post-surgery period and concluded that recessions could be successfully treated with PRF+CAF as well as CTG+CAF.

In a case report recently published, a patient with multiple Miller Class II gingival recessions on the vestibular face of the upper and lower teeth was treated with leukocyte- and platelet-rich fibrin (PRF)+tunnel technique (TT) on his right region and with SCTG+TT on his left region^16^. Four tubes of blood samples (4*10 ml venous blood) were used to treat multiple gingival recession that contains the tooth numbering 11-12-13-14 and SCTG technique with TT to treat the teeth numbered at 21-22-23-24. Pazmiño, et al.^16^ (2017) stated that they chose the TT technique because of the versatility, predictability of success and the little keratinized gingiva. However, CAF technique was used in our study because of the adequate KTT on the apical part of the recessions. This report has a clinical evaluation of 45 days after surgery. Despite the short follow-up period, the author concluded that both CTG and PRF techniques brought a significant quantity of root coverage (90%). The success of this study in PRF+TT technique may be explained by the use of a large amount of PRF clots. However, the study requires long-term evaluation.

PRF membrane homogeneity and the amount of platelet concentrate may be critical for the results^15^. The results of another clinical study, in which PRF membranes+CAF procedure was performed, showed results opposite to our study^3^. In that study, RC was 76.1±17.7% and 88.2±16.9% at 3 months and 80.7±14.7% and 91.5±11.4% at 6 months after surgery for the test group, in which the PRF membranes+CAF technique was used, and for the control group, in which the CAF technique only was used, respectively. Complete root coverage was obtained in 52.23% of the recession in the test group and in 74.62% of the recession in the control group. However, KTT gain was higher in the test group.

Researchers used CTG and PRF membranes for GR and various results were reported in studies in which the CTG+PRF combination was used or where the CTG and PRF were compared.

In a different study, in which groups were prepared in the same manner, autologous platelet-rich fibrin (PRF) and CTG were used to treat multiple adjacent gingival recessions that consisted of two or three teeth as in our study^23^. Despite the similarity in the treatment technique of studies, there were two test groups in our study (2PRF+CAF and 4PRF+CAF) and there was one test group (L-PRF+CAF) in the study conducted by Tunali, et al.^23^ (2015). Although two or four layers of PRF membranes were used in test groups of our study, decreases in RD values were not only statistically significant in our study, but also in the other study. In the study by Tunali, et al.^23^ (2015) initial gingival recession values were deeper compared to our study. The author stated that the significant decreased RD values for test groups may be explained due to the deep defects that can help the occurrence of statistical difference. In such recent study, CAL gain and increase in KGW values between test and control groups were similar. However, CAL gain in the 4PRF+CAF and CTG+CAF groups was higher than in the 2PRF+CAF group, and KTT in the CTG+CAF group was significantly higher than in our test groups. In our study, the parallel design trial was used because of three study groups. Tunali, et al.^23^ (2015) treated multiple bilateral defects, but the author complained due to the limited number of patients.

In other recent study, PRF membranes treated with CTG in test groups were only compared with the CTG procedure in control groups^13^. The results of this study show a statistically significant difference between the groups, such as RC and tissue thickness at 6 months.

Our clinical trial is a study of parallel design. The small number of patients in split-mouth design trials should be considered as a limitation of this study. In our study, clinical measurements and defect-related characteristics were similar in all groups at baseline, and there were more recession defects than in split-mouth studies.

In this study, the RD and RW values decreased the most in the first month in all groups. However, RD values increased again at 6 months in 2PRF+CAF and 4PRF+CAF, and the increase was statistically significant in 2PRF+CAF but not significant in 4PRF+CAF. RD values continued to decrease at 3 and 6 months compared with baseline, but this decrease was not statistically significant in the control group. Changes in RD values in the control group were associated with graft maturation and “creeping attachment”^12^. Changes in RD values in the test groups were associated with resorption of PRF membranes, a decrease in the amount of platelet and growth factors, and the movement of the flap in the apical direction^6^. This may be because the initial amount of PRF membranes and the decrease of the RD values were not statistically significant in 4PRF+CAF.

## Conclusions

The results of the GR treatment procedure in which different amounts of PRF membranes were applied in test groups were compared, and better results were obtained when the maximum possible amount was used. Additionally, closure of the root surface was preserved significantly longer in the follow-up period in these patients. The CAF+PRF membrane technique may be used as an alternative to the CAF+CTG procedure when PRF membranes are adequately used, and more successful results can be achieved with long-term follow-up studies.
